# 18F-Fluorodeoxyglucose Positron Emission Tomography of Head and Neck Cancer: Location and HPV Specific Parameters for Potential Treatment Individualization

**DOI:** 10.3389/fonc.2022.870319

**Published:** 2022-06-08

**Authors:** Sebastian Zschaeck, Julian Weingärtner, Elia Lombardo, Sebastian Marschner, Marina Hajiyianni, Marcus Beck, Daniel Zips, Yimin Li, Qin Lin, Holger Amthauer, Esther G. C. Troost, Jörg van den Hoff, Volker Budach, Jörg Kotzerke, Konstantinos Ferentinos, Efstratios Karagiannis, David Kaul, Vincent Gregoire, Adrien Holzgreve, Nathalie L. Albert, Pavel Nikulin, Michael Bachmann, Klaus Kopka, Mechthild Krause, Michael Baumann, Joanna Kazmierska, Paulina Cegla, Witold Cholewinski, Iosif Strouthos, Klaus Zöphel, Ewa Majchrzak, Guillaume Landry, Claus Belka, Carmen Stromberger, Frank Hofheinz

**Affiliations:** ^1^ Department of Radiation Oncology, Berlin Institute of Health, Charité – Universitätsmedizin Berlin, Corporate Member of Freie Universität Berlin, Humboldt-Universität zu Berlin, Berlin, Germany; ^2^ Berlin Institute of Health (BIH), Berlin, Germany; ^3^ Department of Radiotherapy and Radiation Oncology, Faculty of Medicine and University Hospital Carl Gustav Carus, Technische Universität Dresden, Dresden, Germany; ^4^ German Cancer Consortium (DKTK), Partner Site Dresden, and German Cancer Research Center (DKFZ) Heidelberg, Germany, Germany; ^5^ OncoRay – National Center for Radiation Research in Oncology, Faculty of Medicine and University Hospital Carl Gustav Carus, Technische Universität Dresden, Helmholtz-Zentrum Dresden – Rossendorf, Dresden, Germany; ^6^ Department of Radiation Oncology, University Hospital, Ludwig-Maximilians-University (LMU) Munich, Munich, Germany; ^7^ German Cancer Consortium (DKTK), Partner Site Munich, Munich, Germany; ^8^ German Cancer Consortium (DKTK), Partner Site Tübingen, and German Cancer Research Center (DKFZ) Heidelberg, Germany, Germany; ^9^ Department of Radiation Oncology, University Hospital and Medical Faculty, Eberhard Karls University Tübingen, Tübingen, Germany; ^10^ Department of Radiation Oncology, Xiamen Cancer Center, The First Affiliated Hospital of Xiamen University, Xiamen, China; ^11^ Department of Nuclear Medicine, Berlin Institute of Health, Charité – Universitätsmedizin Berlin, Corporate Member of Freie Universität Berlin, Humboldt-Universität zu Berlin, Berlin, Germany; ^12^ Institute of Radiooncology – OncoRay, Helmholtz-Zentrum Dresden - Rossendorf, Dresden, Germany; ^13^ National Center for Tumor Diseases (NCT), Partner Site Dresden, Germany: German Cancer Research Center (DKFZ), Heidelberg, Germany; ^14^ Faculty of Medicine and University Hospital Carl Gustav Carus, Technische Universität Dresden, Dresden, Germany; ^15^ Helmholtz Association/Helmholtz-Zentrum Dresden - Rossendorf (HZDR), Dresden, Germany; ^16^ Institute of Radiopharmaceutical Cancer Research, Helmholtz-Zentrum Dresden-Rossendorf, Dresden, Germany; ^17^ Department of Nuclear Medicine, Faculty of Medicine and University Hospital Carl Gustav Carus, Dresden, Germany; ^18^ Department of Radiation Oncology, German Oncology Center, European University Cyprus, Limassol, Cyprus; ^19^ Radiation Oncology Department, Leon Bérard Cancer Center, Lyon, France; ^20^ Department of Nuclear Medicine, University Hospital, Ludwig-Maximilians-University (LMU) Munich, Germany; ^21^ German Cancer Research Center (DKFZ), Heidelberg, Germany; ^22^ Electroradiology Department, University of Medical Sciences, Poznan, Poland; ^23^ Radiotherapy Department II, Greater Poland Cancer Centre, Poznan, Poland; ^24^ Department of Nuclear Medicine, Greater Poland Cancer Centre, Poznan, Poland; ^25^ Department of Nuclear Medicine, Klinikum Chemnitz gGmbH, Chemnitz, Germany; ^26^ Department of Head and Neck Surgery, Poznan University of Medical Sciences, Greater Poland Cancer Centre, Poznan, Poland

**Keywords:** head and neck squamous cell carcinoma (HNSCC), fluorodeoxyglucose positron emission tomography (FDG PET), radiotherapy, metabolic tumor volume (MTV), standardized uptake value (SUV)

## Abstract

**Purpose:**

18F-fluorodeoxyglucose positron emission tomography (FDG-PET) is utilized for staging and treatment planning of head and neck squamous cell carcinomas (HNSCC). Some older publications on the prognostic relevance showed inconclusive results, most probably due to small study sizes. This study evaluates the prognostic and potentially predictive value of FDG-PET in a large multi-center analysis.

**Methods:**

Original analysis of individual FDG-PET and patient data from 16 international centers (8 institutional datasets, 8 public repositories) with 1104 patients. All patients received curative intent radiotherapy/chemoradiation (CRT) and pre-treatment FDG-PET imaging. Primary tumors were semi-automatically delineated for calculation of SUV_max_, SUV_mean_, metabolic tumor volume (MTV) and total lesion glycolysis (TLG). Cox regression analyses were performed for event-free survival (EFS), overall survival (OS), loco-regional control (LRC) and freedom from distant metastases (FFDM).

**Results:**

FDG-PET parameters were associated with patient outcome in the whole cohort regarding clinical endpoints (EFS, OS, LRC, FFDM), in uni- and multivariate Cox regression analyses. Several previously published cut-off values were successfully validated. Subgroup analyses identified tumor- and human papillomavirus (HPV) specific parameters. In HPV positive oropharynx cancer (OPC) SUV_max_ was well suited to identify patients with excellent LRC for organ preservation. Patients with SUV_max_ of 14 or less were unlikely to develop loco-regional recurrence after definitive CRT. In contrast FDG PET parameters deliver only limited prognostic information in laryngeal cancer.

**Conclusion:**

FDG-PET parameters bear considerable prognostic value in HNSCC and potential predictive value in subgroups of patients, especially regarding treatment de-intensification and organ-preservation. The potential predictive value needs further validation in appropriate control groups. Further research on advanced imaging approaches including radiomics or artificial intelligence methods should implement the identified cut-off values as benchmark routine imaging parameters.

## Introduction

In head and neck squamous cell carcinomas (HNSCC) beside computed tomography (CT), positron emission tomography (PET) with the radiotracer 18f-fluorodeoxyglucose (FDG) is frequently used for tumor staging and treatment planning in clinical routine (1). Various PET parameters have been investigated regarding their prognostic value in HNSCC. One requirement of imaging parameters is that these parameters bear independent prognostic value compared to established clinical parameters. In FDG-PET, metabolic tumor volume (MTV), maximum and mean standardized uptake value (SUV_max_ and SUV_mean_), and the derived parameter total lesion glycolysis TLG (defined as MTV × SUV_mean_) can be seen as standard parameters that can be easily evaluated in clinical routine. Currently, the prognostic impact of these parameters is not well defined, especially in biologically heterogeneous sub-groups of HNSCC. A meta-analysis of studies investigating the prognostic value of pre-therapeutic FDG-PET in patients treated with definitive chemoradiation (CRT) reported that only MTV has significant prognostic impact on patients´ outcome (2). However, outcome parameters were only available for a minority of patients. Especially concerning the important endpoint loco-regional control (LRC), only four out of 25 studies included sufficient information. Additionally, the included studies used different tumor segmentation methods, therefore MTV delineation can differ considerably and the MTV values cannot be directly compared between studies. These limitations hamper any valid conclusions regarding the prognostic value of FDG-PET in HNSCC treated with definitive CRT. The aim of this study was to perform a multicenter analysis of original FDG-PET data from HNSCC patients treated with definitive CRT. All images were centrally analyzed by the same observer with the same software and semi-automatic delineation methods. Results of a small subgroup of patients with nasopharyngeal cancer (NPC) have already been published, here we report a larger cohort with additional NPC patients and all other tumor locations (3).

## Patients and Methods

### Patients

Inclusion criteria for this study were: histologically confirmed HNSCC without evidence of distant metastases, definitive radiotherapy or CRT with curative intent, and availability of pre-treatment FDG-PET. We analyzed PET images and patient data from one Chinese and seven European centers plus additional images and patient data from the cancer imaging archive (4), in particular: Head-Neck-PET-CT, QIN-HEADNECK, HNSCC, TCGA-HNSC, Head-Neck-Radiomics-HN1 (5–12). TNM classification was bases on American Joint Committee on cancer staging manual version number seven.

### Imaging

Details on the imaging of the patients from public databases can be found in the original publications cited above. Patients from Berlin, Xiamen, Dresden, Brussels, Tuebingen, Limassol, Poznan and Munich received hybrid-imaging (usually PET-CT) with the following equipment: Gemini TF 16 (Philips Medical Systems, Cleveland, OH, USA), Discovery STE (General Electric Medical Systems, Milwaukee, WI, USA), Biograph 16 PET/CT scanner (Siemens Medical Solutions Inc., Knoxville, TN), Gemini TF PET-CT (Philips Medical Systems, Cleveland, OH, USA), Biograph mCT (Siemens Healthineers), Discovery IQ (General Electric Medical Systems, Milwaukee, WI, USA), Gemini TF TOF 16 (Philips Healthcare Inc., Andover, MA) and Discovery (General Electric Medical Systems, Milwaukee, WI, USA)/Biograph (Siemens Medical Solutions Inc., Knoxville, TN), respectively.

### Treatment

All patients received primary radiotherapy with curative intent. Radiotherapy was performed as three-dimensional, intensity modulated or volumetric modulated treatment. Prescribed radiation doses ranged between 66 and 77 Gray (Gy). In most cases radiotherapy was combined with simultaneous chemotherapy or cetuximab. 804 patients received concomitant systemic therapy. Most patients with individual data on chemotherapy received platinum-based CRT regimes (371 of 439 patients; 85%), 165 patients received radiotherapy only (15%) and 135 patients (12%) had no information available on concomitant therapy. Commonly only patients with early stage disease were treated by radiotherapy only, while CRT was prescribed in locally advanced stages.

### Image Analysis

The metabolically active part of the primary tumor was delineated in the PET data by a semi-automatic algorithm based on adaptive thresholding considering the local background (13, 14).

Manual delineation was only performed in case of low or diffuse tracer accumulation. In case of lacking tracer accumulation the voxel with highest activity within the primary tumor site (i.e. with the highest SUV_max_ uptake) was contoured as a single voxel for further analyses, this was the case in 30 patients (2.7%). This approach was chosen to avoid bias by excluding all patients without significant FDG uptake from the analyses. For the resulting regions of interest (ROIs), the metabolic active tumor volume (MTV), maximum and mean standardized uptake value (SUV_max_ and SUV_mean_), and the total lesion glycolysis (MTV × SUV_mean_, TLG) were computed. Delineation was performed by an experienced radiation oncologist (SZ) and verified by an experienced Nuclear Medicine physician (KZ). ROI definition and ROI analyses were performed using the software ROVER version 3.0.41 (ABX GmbH, Radeberg, Germany).

### Statistical Analyses

Survival analysis was performed with respect to event free survival (EFS), overall survival (OS), locoregional control (LRC), and freedom from distant metastases (FFDM). The association of endpoints with clinical and quantitative PET parameters was analyzed using univariate and multivariate Cox proportional hazard regression in which the PET parameters were included as metric parameters. Parameters were further analyzed in univariate Cox regression using binarized PET parameters. The cut-off values were calculated by minimizing the p-value in univariate Cox regression as described in (15). The optimal cut-off was determined separately for EFS, OS, LRC, and FFDM. Cut-off values leading to p<0.05 were tested for stability (i.e., sensibility of the prognostic value against variation of the cut-off value). In this test, the range of cut-off values still leading to a significant effect in univariate analysis was computed by successively decreasing/increasing the cut-off value (starting at the optimal value) and repeating univariate Cox regression. Probability of event occurrence was computed and rendered as Kaplan–Meier curves. Statistical significance was defined as a p-value of less than 0.05. Statistical analysis was performed with the R language and environment for statistical computing version 4.0.5 (16).

For validation of previously published cut-off values, all 25 studies included in the meta-analysis were searched for reported significant cut-off values, published endpoints, and tumor locations (2). **Supplementary Table 1** summarizes these data (only analyses of the primary tumor parameters were considered).

## Results

**Figure 1:**
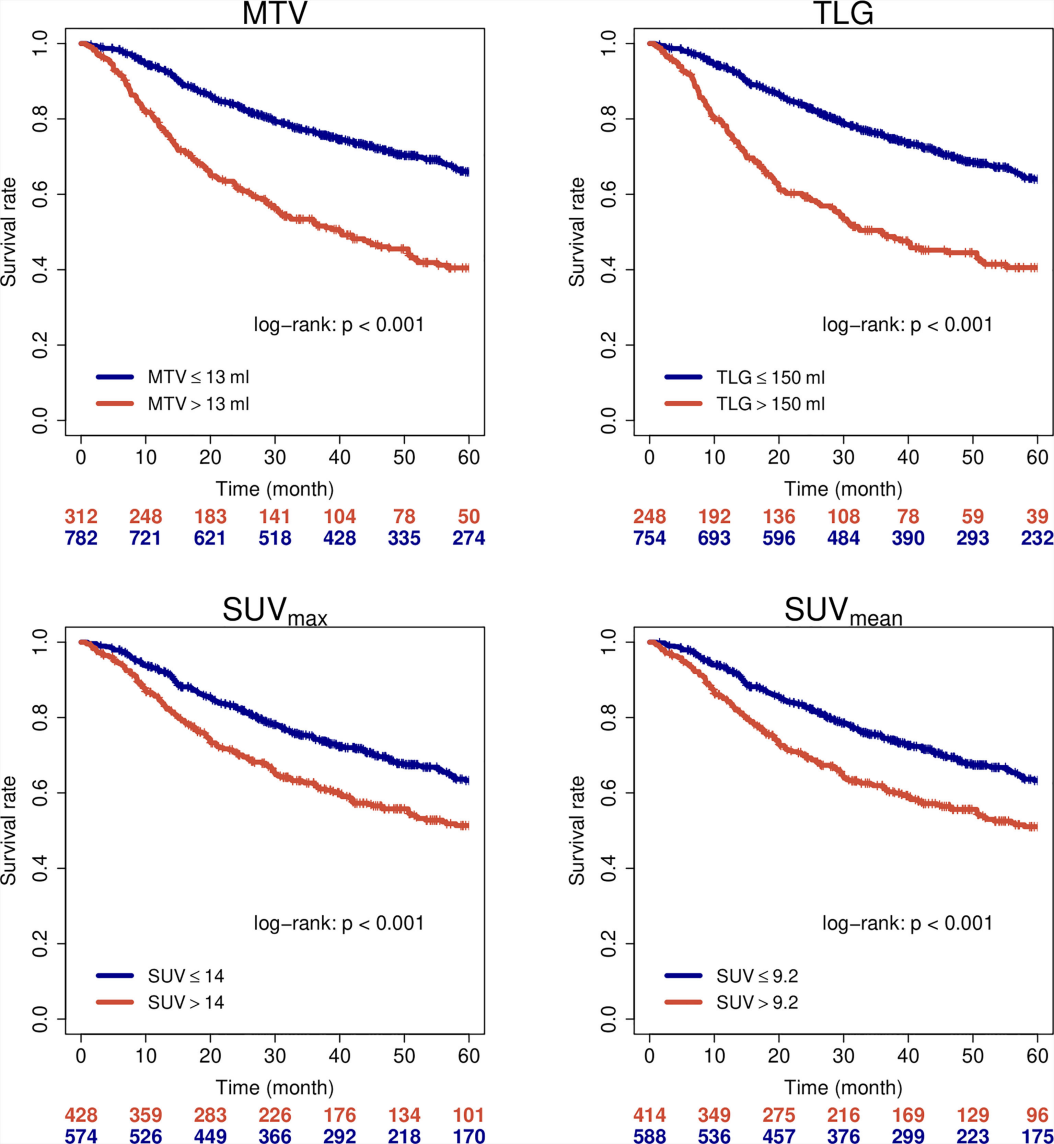
Overall survival of all patients when stratified by PET parameters.

**Figure 2:**
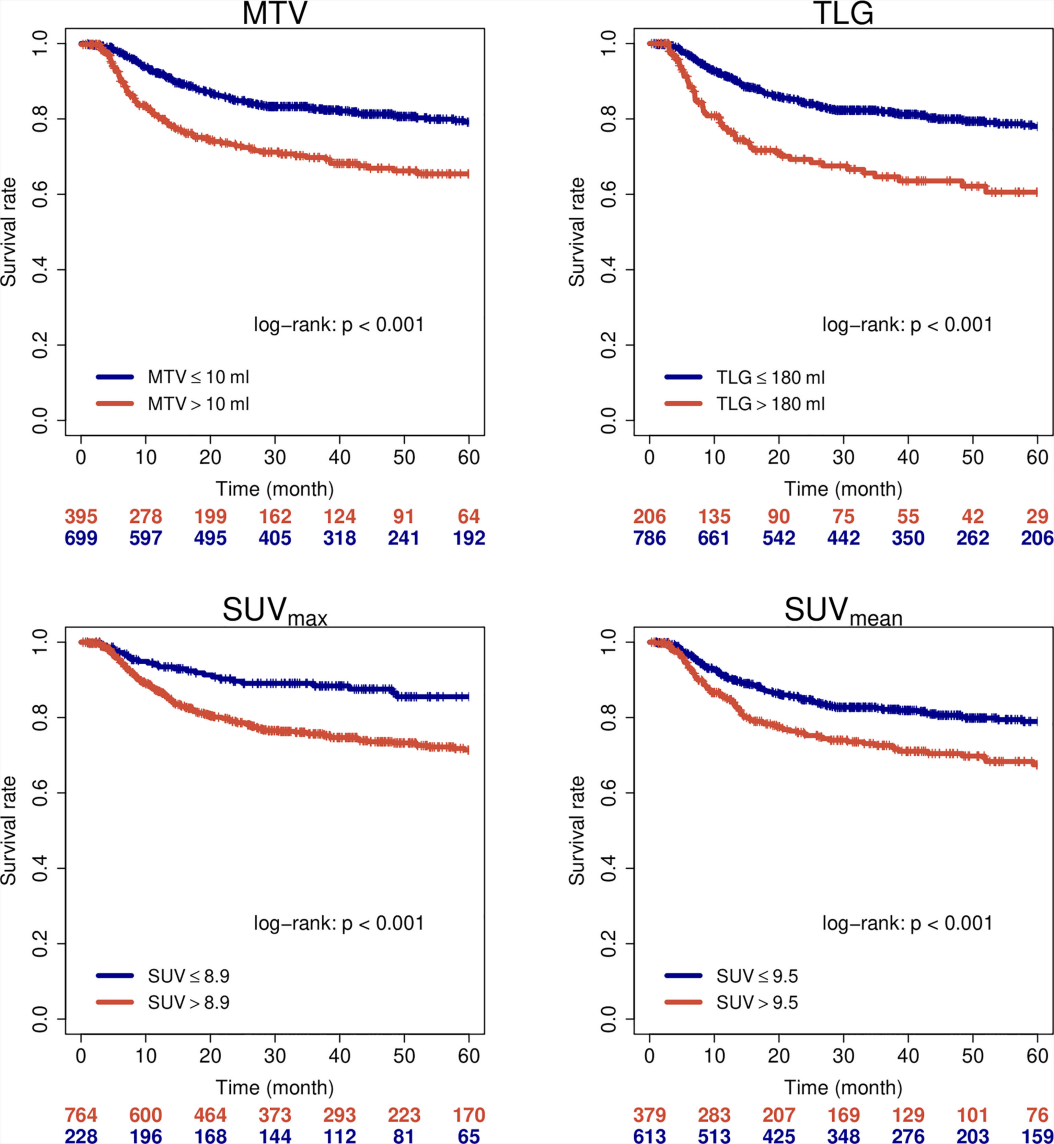
Loco-regional control of all patients when stratified by PET parameters.

**Figure 3:**
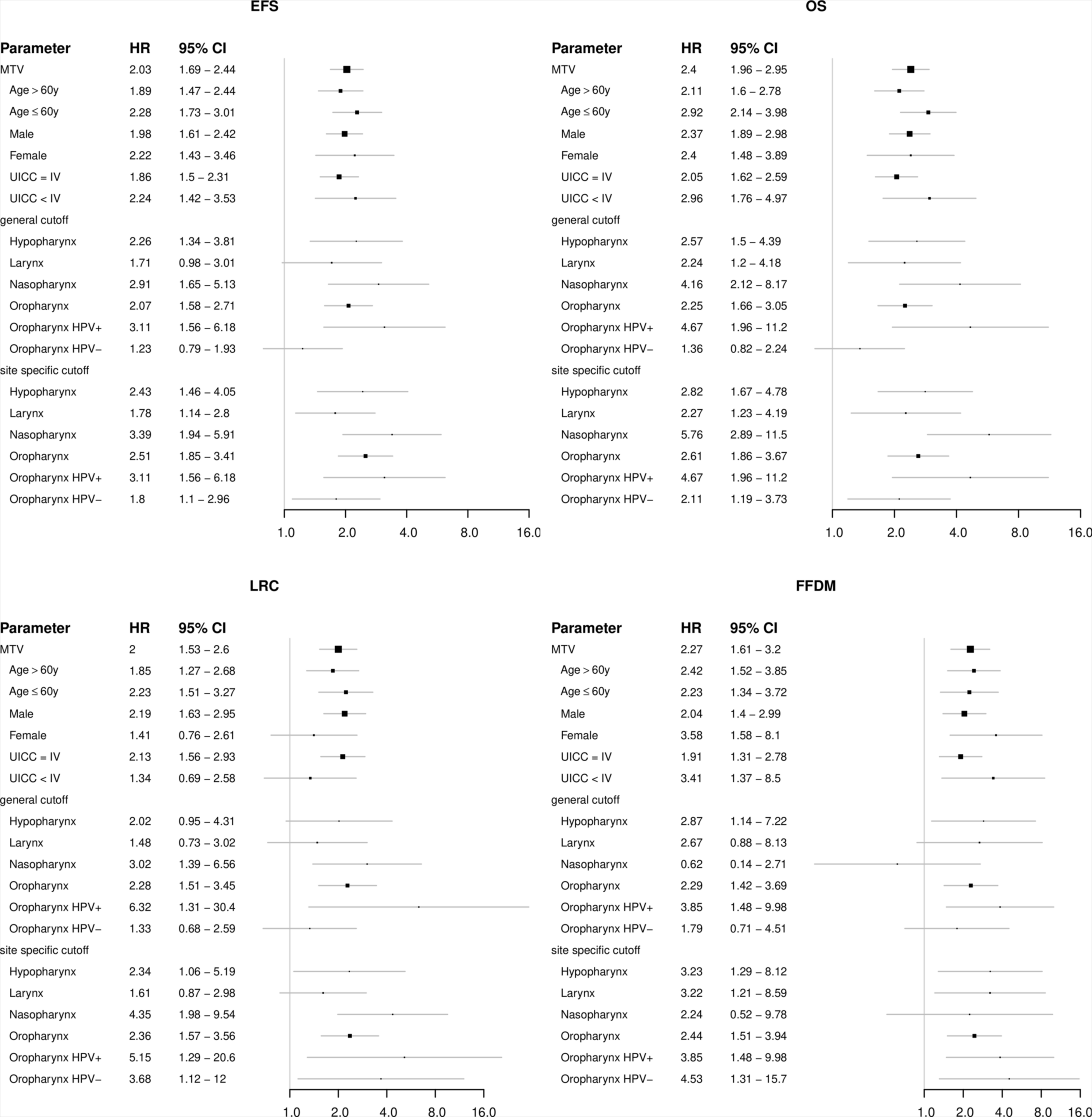
Forest plots showing the prognostic value of the FDG-PET parameter metabolic tumor volume (MTV) in subgroups of patients regarding the clinical endpoints EFS, OS, LRC and FFDM.

**Figure 4:**
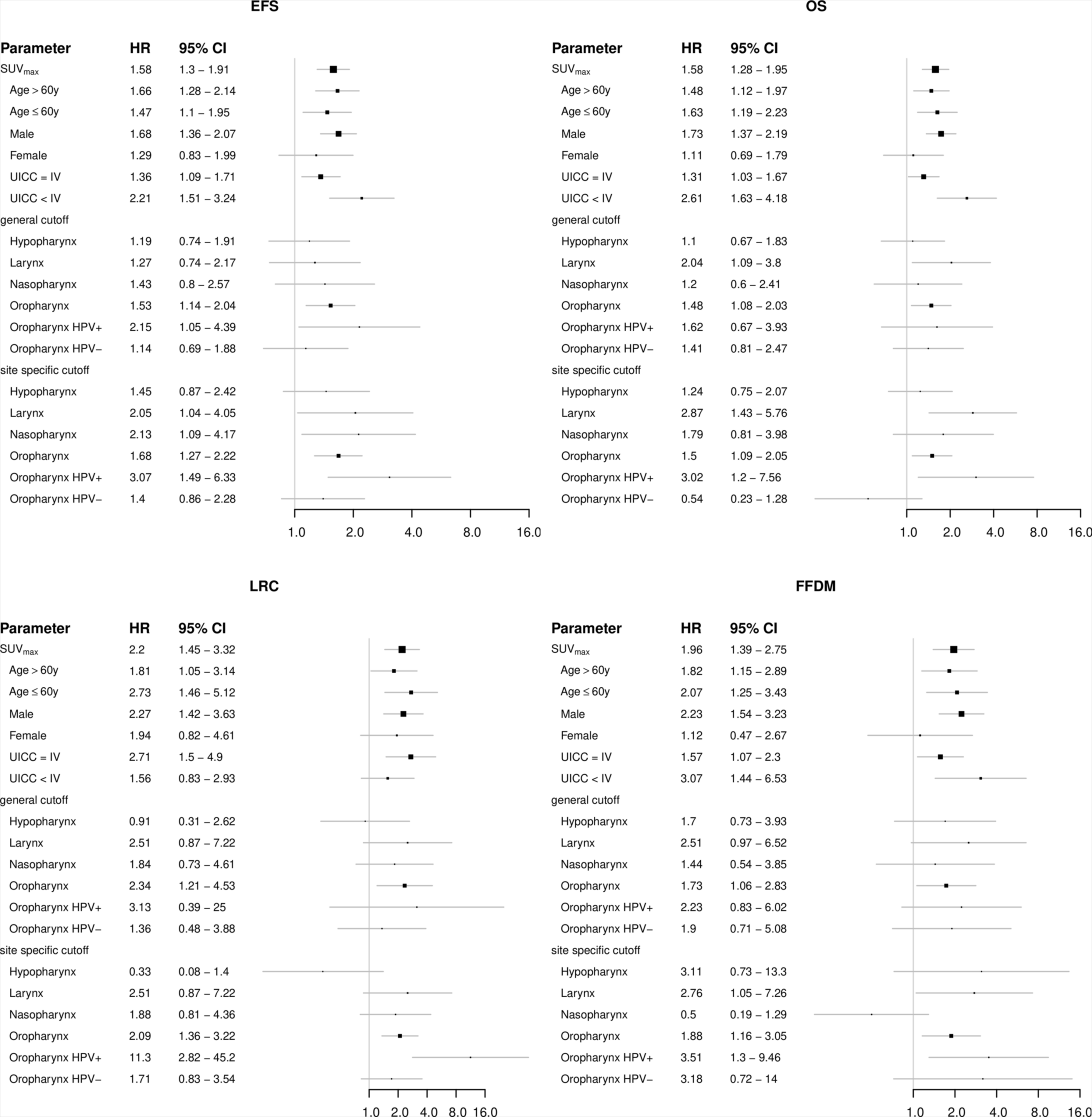
Forest plots showing the prognostic value of the FDG-PET parameter maximum standardized uptake value (SUV_max_) in subgroups of patients regarding the clinical endpoints EFS, OS, LRC and FFDM.

**Figure 5:**
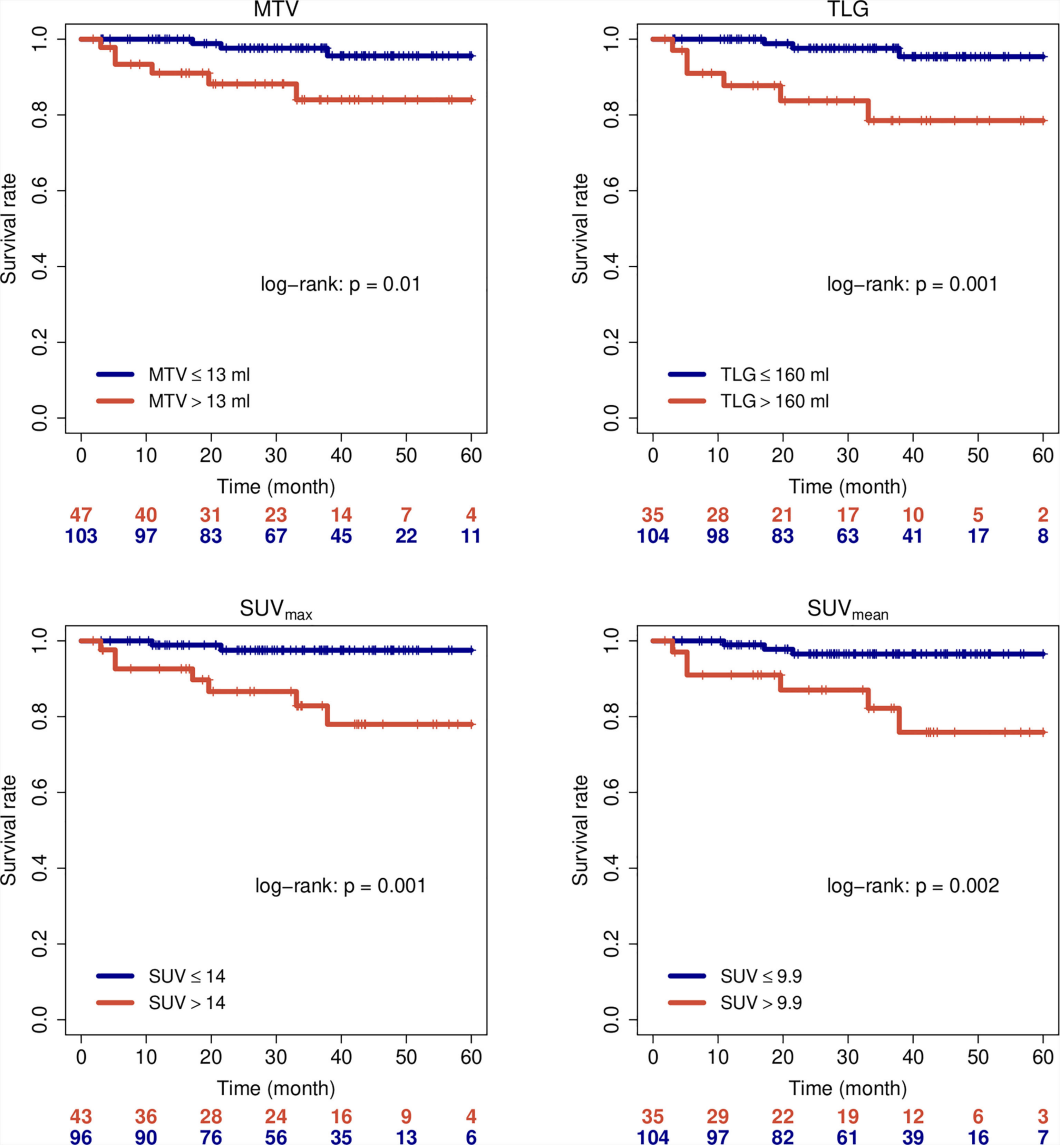
Loco-regional control of HPV-positive oropharyngeal carcinomas when stratified by PET parameters.

1104 patients with individual patient and outcome data and original PET images for analysis were included in this study. **Supplementary Table 2** summarizes available clinical data of all 16 cohorts. Median age of patients was 60 years and 79% of patients were male. The vast majority of patients presented locally advanced stages of HNSCC (87% > UICC stage II) and oropharynx (OPC) was the most frequent primary tumor location (51%). Detailed patient characteristics are reported in **Supplementary Table 3**. In the whole cohort, median MTV, TLG, SUV_max_ and SUV_mean_ were 7.0 ml, 61.6 ml, 13.0 and 8.4, respectively. Details and distribution of the parameters are reported in **Supplementary Table 4**. There were significant differences of PET parameters depending on tumor location. Broadly speaking, tumors located within the oral cavity (OCC) had higher values and larynx carcinomas (LC) showed lower values of some parameters. Details on the distribution can be found in **Supplementary Figure 1** and comparison between groups is shown in **Supplementary Table 5**. Since tumor location is a known prognostic factor in HNSCC, we checked for prognostic relevance of this parameter in this cohort (see **Supplementary Figure 2**) and added the information in uni- and multivariate cox regression analyses. When analyzing oncological outcome of the whole cohort, all PET parameters showed a significant association with all investigated clinical endpoints (EFS, OS, LRC and FFDM). Details are reported in **Table 1**. Note that not all parameters/endpoints were available for all patients. Patients with missing information were excluded in the respective analysis. The number of included patients is listed in column ‘N’ in **Table 1**. The results are more or less unchanged when patients with missing information are exclude completely (see **Supplementary Table 6**). Upon multivariate testing, MTV showed a robust association with EFS, OS and LRC, while SUV_max_ showed the highest association with FFDM. Details are reported in **Table 2**. Only those patients were included for which all information on all analyzed parameters/endpoints was available. The number of included patients is indicated at the top of the corresponding part of **Table 2**. Binarization and cutoff-stability testing of PET parameters revealed that MTV, TLG, and, in some cases, SUV_max_ are able to significantly discriminate between risk groups across a broad range of values (**Supplementary Tables 7**, **8**). Several previously published cut-off values were successfully validated (**Supplementary Table 9**). **Figures 1**, **2** show Kaplan-Meier estimates for patients stratified according to PET parameters with the endpoints OS and LRC. Figures for EFS and FFDM are shown in **Supplementary Figures 3, 4**. Since tumor location and HPV status have a strong influence on the outcome of patients, PET parameters were optimized for each tumor subtype. **Figures 3**, **4** show Forest plots of the prognostic significance of PET parameters in different subgroups including primary tumor site for PET parameters MTV and SUV_max_. MTV seems to bear the highest prognostic value especially in younger patients and in patients with NPC, which is partly correlated (average age of NPC patients in this cohort 52.4 years versus 61.1 years for non-NPC HNSCC, p < 0.001). Additionally, NPC did show a different behavior regarding FFDM compared to other locations (the only location with decreased risk of FFDM with increased MTV or SUV_max_ although not reaching statistical significance). A surprising finding was the very strong association of SUV_max_ with LRC in HPV-positive (HPV+) OPC. **Figure 5** shows Kaplan-Meier estimates for HPV-positive OPC stratified according to the investigated PET parameters. The SUV_max_ cut-off of 14 was able to identify patients with excellent LRC after CRT/radiotherapy. While in general MTV seems to be an important risk factor regarding LRC, this does not seem to be the case in LC. Also other PET parameters did not show a convincing association in LC (**Supplementary Figure 5**).

**Table 1:** Univariate cox regression analyses with respect to EFS, OS, LRC and FFDM.

Parameter	EFS				OS			
	N	HR	95% CI	p-value	N	HR	95% CI	p-value
Sex male	1078	1.17	0.93 – 1.47	0.18	1094	1.17	0.91 – 1.51	0.22
Age > 60y	1078	1.51	1.26 – 1.81	<0.001	1078	1.57	1.28 – 1.93	<0.001
T-stage > 2	1074	1.93	1.58 – 2.36	<0.001	1090	2.17	1.72 – 2.75	<0.001
N-stage > 0	1074	1.3	1.03 – 1.64	0.025	1090	1.74	1.31 – 2.31	<0.001
UICC-stage > III	1047	1.48	1.2 – 1.82	<0.001	1063	1.92	1.49 – 2.46	<0.001
HPC+oral cavity	1078	2.48	2.02 – 3.05	*<*0.001	1094	2.98	2.39 – 3.72	*<*0.001
Chemotherapy NO	965	1.15	0.92 – 1.44	0.22	969	1.24	0.96 – 1.61	0.099
MTV	1078	1.02	1.02 – 1.02	<0.001	1094	1.02	1.02 – 1.02	<0.001
TLG	986	1.002	1.001 – 1.002	<0.001	1002	1.002	1.001 – 1.002	<0.001
SUV_max_	986	1.02	1.01 – 1.03	<0.001	1002	1.02	1.01 – 1.04	<0.001
SUV_mean_	986	1.03	1.01 – 1.05	0.0075	1002	1.03	1.01 – 1.06	0.005
**Parameter**	**LRC**				**FFDM**			
	**N**	**HR**	**95% CI**	**p-value**	**N**	**HR**	**95% CI**	**p-value**
Sex male	1094	1.005	0.728 – 1.388	0.97	1061	1.35	0.87 – 2.09	0.18
Age > 60y	1073	1.24	0.95 – 1.62	0.11	1060	1.4	1.01 – 1.96	0.047
T-stage > 2	1087	1.98	1.46 – 2.68	<0.001	1057	1.82	1.26 – 2.63	0.002
N-stage > 0	1087	1.02	0.74 – 1.42	0.89	1057	3.41	1.84 – 6.3	<0.001
UICC-stage > III	1060	1.36	1.01 – 1.83	0.046	1030	2.24	1.47 – 3.41	<0.001
HPC+oral cavity	1094	2.34	1.73 – 3.18	<0.001	1061	2.19	1.48 – 3.24	<0.001
Chemotherapy NO	960	0.994	0.713 – 1.386	0.97	961	2.01	1.25 – 3.23	0.004
MTV	1094	1.02	1.02 – 1.03	<0.001	1061	1.02	1.01 – 1.02	<0.001
TLG	992	1.002	1.001 – 1.002	<0.001	969	1.001	1.001 – 1.002	<0.001
SUV_max_	992	1.03	1.01 – 1.04	0.011	969	1.03	1.01 – 1.05	0.015
SUV_mean_	992	1.03	1 – 1.06	0.027	969	1.04	1 – 1.07	0.05

PET parameters were included as metric parameters. Column ‘N’ shows the number of included patients in the respective analysis.

**Table 2:** Multivariate cox regression analyses with respect to EFS, OS, LRC and FFDM.

Parameter	EFS (n=955)			OS (n=971)		
	HR	95% CI	P-value	HR	95% CI	P-value
Age	1.5	1.23 –1.82	*<*0.001	1.66	1.34–2.07	*<*0.001
T stage		–			–	
UICC stage	1.2	0.957–1.51	0.11	1.54	1.17–2.03	0.002
Chemotherapy		–			–	
HPC + Oral Cavity	2.55	2.06–3.16	*<*0.001	3.1	2.46–3.92	*<*0.001
MTV	1.86	1.51–2.29	*<*0.001	2.27	1.8–2.87	*<*0.001
SUV_max_	1.31	0.996 –1.73	0.053	1.14	0.91–1.42	0.26
**Parameter**		**LRC (n=988)**		**FFDM (n=911)**		
	**HR**	**95% CI**	**P-value**	**HR**	**95% CI**	**P-value**
Age		–		1.4	0.985–1.98	0.061
T stage	1.24	0.858 – 1.79	0.25		–	
UICC stage		–		1.6	1.02–2.51	0.041
Chemotherapy		–		1.48	0.904–2.43	0.12
HPC + Oral Cavity	2.31	1.68 – 3.18	*<*0.001	1.78	1.17–2.71	0.007
MTV	1.79	1.3–2.45	*<*0.001	1.83	1.25–2.68	0.002
SUV_max_	1.56	1.01–2.41	0.043	1.44	1.01–2.07	0.047

PET parameters were included as metric parameters. The number of included patients is shown at the top of the corresponding part of the table.

## Discussion

Based on a plethora of mostly retrospective single-center studies, FDG-PET parameters are considered significant prognostic and potentially predictive parameters for response to CRT in HNSCC. This is reflected by the use of high FDG uptake volumes for dose escalation in several trials (17–20). In a recent review article Clausen and colleagues argued that regarding its prognostic value FDG is beyond the exploratory phase; at the same time they found evidence for publication bias with potential overestimation of the prognostic effect size of FDG parameters in HNSCC (21). Their analysis, which was not based on individual patient data, included 1704 HNSCC patients from 20 studies with a median sample size of only 58 patients. Given the distinct biological features and prognosis of HNSCC subtypes, this implies that subgroup analyses are not useful in the majority of small sample size publications.

Here we report the, to our knowledge, largest individual patient and imaging based analyses of HNSCC with pre-treatment FDG-PET and primary CRT/radiotherapy. Our analyses confirm a moderate association of several PET parameters with clinical outcome of patients in the whole cohort. At the same time, considerable differences regarding primary tumor location and HPV status were found. This is an important observation for future aims to personalize radio-oncological treatment by the implementation of PET parameters. In early stage OPC, radiotherapy and transoral robotic surgery showed comparable quality of life and outcome, but with different toxicity profiles in a randomized phase-II study (22, 23). Although high-level evidence is lacking for other and more advanced HNSCC, CRT and primary surgery are often considered similar efficient primary treatment approaches. Since toxicity and late side effects are considerable in the head and neck region, biomarkers to individualize treatment are urgently needed for these patients.

LC and HPV positive OPC are probably two of the tumor locations with the strongest need for parameters to individualize treatment, i.e. schedule patients for primary surgery or CRT depending on the probability of LRC. Our analyses revealed that FDG-PET does not deliver convincing information in LC, however in HPV positive OPC, several PET parameters show a very high discriminatory ability. Even the most easily obtainable parameter SUV_max_, assessed during every clinical routine PET scan, seems to be very well suited to select low-risk patients that could potentially be treated within dose de-escalation trials. To our opinion this is an unexpected finding especially in a multi-center analysis, since SUV parameters are known to be prone to several potential errors. They are uptake-time-dependent and time after injection differs considerably in routine care patients. Furthermore SUV is susceptible to scanner calibration errors and the correlation between systemic tracer distribution and body weight is only weak, adding additional variability in common SUV calculations. Especially in multicenter analyses, this can make quantitative comparison of SUV difficult and our group was able to show that the uptake time normalized ratio of tumor SUV and blood SUV (SUR) is superior to tumor SUV alone regarding correlation with glucose uptake of lesions, but also regarding outcome discrimination (24–27). However, determination of blood SUV requires a PET/CT scan of the thorax. Some of the included patients had missing corresponding CT scans or PET examination limited to the upper thorax and head and neck region. Therefore, calculation of SUR was not possible in the whole cohort, but is subject of ongoing research in a subgroup of patients with available imaging information. The strong prognostic value of SUV_max_ in this cohort of HPV positive OPC despite its methodological limitations, might be due to the relatively high cutoff value. Given the increasing incidence of HPV positive OPC in combination with the relatively good prognosis, further individually tailored treatment is an urgent medical need (28). Several phase-II studies reported promising outcome results with CRT dose de-escalation to 60 Gy (29, 30). Another phase-II study was able to show that normo-fractionated dose de-escalation down to 50 Gy is feasible after prior selection of patients with favorable biology using induction chemotherapy (31). However, current evidence does not suggest any benefit by induction chemotherapy for OPC patients (32). Therefore, other biomarkers, as our identified PET parameter, would be ideal candidates to guide future treatment de-escalation.

Our study has several limitations such as the retrospective nature of the data and partly missing information, especially in data from public repositories. Most obviously, this affects the HPV status of oropharyngeal carcinomas, which was not available for a relevant number of patients. Furthermore, the TNM staging classification was not according to the most current version number eight. There were substantial modifications from version seven to version eight, with emphasis on HPV positive oropharynx carcinomas (33). This is a major drawback when comparing the data with other current HNSCC data and should be considered when interpreting the data. Nonetheless, most of the basic parameters, including the important endpoints LRC and OS were available and the current analysis includes by far the largest dataset of FDG-PET from HNSCC patients. Therefore, our analysis can be regarded as reference benchmark for future research on the prognostic value of imaging parameters, i.e. the identified parameters should be considered when establishing novel radiomics and/or AI models for prognostication of HNSCC patients and novel signatures should outperform these parameters regarding patient stratification. This seems to be highly important as a recent analysis has shown that one of the most popular radiomics signatures, that has been independently validated, is highly correlated with tumor volume (34). Our analysis does not only identify promising future applications for standard parameters, but also shows for which tumor subtypes and outcome parameters further research on optimal stratification and treatment personalization is warranted. For example, standard PET parameters of primary tumors have not shown convincing results regarding the prediction of distant metastases. Analysis of affected lymph nodes and/or more sophisticated image analyses by convolutional neural networks have shown promising results and should probably be further developed to address this important issue (35–37). The same holds true for laryngeal carcinomas, for which some early radiomic analyses reported encouraging results (38, 39).

## Conclusion

Standard FDG-PET parameters bear significant prognostic value in HNSCC treated with radiotherapy/CRT but moderate effect size regarding LRC and FFDM in the entire cohort. Subgroup specific analyses revealed SUV_max_ as a promising parameter to select HPV-positive OPC with excellent outcome after CRT/radiotherapy.

## Data Availability Statement

The original contributions presented in the study are included in the article/**Supplementary Material**. Further inquiries can be directed to the corresponding author.

## Ethics Statement

The studies involving human participants were reviewed and approved by Ethikkomission der Charité, Charité Universitätsmedizin Berlin, Berlin, Germany. The patients/participants provided their written informed consent to participate in this study.

## Author Contributions

Study conception and design: SZ and FH; Drafting of manuscript: SZ and FH; Image processing and analysis: SZ, JW, EL, SM, MH, YL, JKo, KF, EK, VG, AH, PN, JKa, PC, KZ, and FH; Study Investigators: SZ, DZ, QL, HA, ET, JvdH, VB, JKo, VG, NA, MK, MBau, WC, KZ, GL, CB, CS, and FH; Interpretation of data: all authors; Final approval of manuscript: all authors.

## Funding

This work was partly supported by the Berliner Krebsgesellschaft (ZSF201720) and by the German Federal Ministry of Education and Research (BMBF contract 03ZIK42/OncoRay). The funders had no role in the design of the study; the collection, analysis, and interpretation of the data; the writing of the manuscript; and the decision to submit the manuscript for publication.

## Conflict of Interest

In the past 5 years, MBau received funding for his research projects and for educational grants to the University of Dresden by Bayer AG (2016–2018), Merck KGaA (2014-open) and Medipan GmbH (2014–2018). He is on the supervisory board of HI-STEM gGmbH (Heidelberg) for the German Cancer Research Center (DKFZ, Heidelberg) and also member of the supervisory body of the Charité University Hospital, Berlin. As former chair of OncoRay (Dresden) and present CEO and Scientific Chair of the German Cancer Research Center (DKFZ, Heidelberg), he has been or is responsible for collaborations with a multitude of companies and institutions, worldwide. In this capacity, he has discussed potential projects and signed contracts for research funding and/or collaborations with industry and academia for his institute(s) and staff, including but not limited to pharmaceutical companies such as Bayer, Boehringer Ingelheim, Bosch, Roche and other companies such as Siemens, IBA, Varian, Elekta, Bruker, etc. In this role, he was/is also responsible for the commercial technology transfer activities of his institute(s), including the creation of start-ups and licensing. This includes the DKFZ-PSMA617 related patent portfolio [WO2015055318 (A1), ANTIGEN (PSMA)] and similar IP portfolios. MBau confirms that, to the best of his knowledge, none of the above funding sources were involved in the preparation of this paper. In the past 5 years, MK received funding for her research projects by IBA (2016), Merck KGaA (2014-2018 for preclinical study; 2018-2020 for clinical study), Medipan GmbH (2014–2018). She is involved in an ongoing publicly funded (German Federal Ministry of Education and Research) project with the companies Medipan, Attomol GmbH, GA Generic Assays GmbH, Gesellschaft für medizinische und wissenschaftliche genetische Analysen, Lipotype GmbH and PolyAn GmbH (2019–2021). For the present manuscript, MK confirms that none of the above funding sources were involved in the preparation of this paper.

HA declares research grants, travel grants, and lecture fees from Sirtex Medical Europe; HA confirms that none of the above funding sources were involved in the preparation of this paper.

The remaining authors declare that the research was conducted in the absence of any commercial or financial relationships that could be construed as a potential conflict of interest.

The reviewer FH declared a past co-authorship with one of the authors ET to the handling Editor.

## Publisher’s Note

All claims expressed in this article are solely those of the authors and do not necessarily represent those of their affiliated organizations, or those of the publisher, the editors and the reviewers. Any product that may be evaluated in this article, or claim that may be made by its manufacturer, is not guaranteed or endorsed by the publisher.
