# Supplementary material for: 18F-Fluorodeoxyglucose Positron Emission Tomography of Head and Neck Cancer: Location and HPV Specific Parameters for Potential Treatment Individualization
Source: Front Oncol. 2022 Jun 8;12:870319. doi: 10.3389/fonc.2022.870319 (PMC9213669; doi:10.3389/fonc.2022.870319)

Supplementary Material

**Supplementary table 1:** Published cut-off values according to^5^:

| **study** | **number of patients** | **tumor location** | **identified L(R)C discriminators** | **identified OS discriminators** | **identified DFS /DSS discriminators** | **identified FFDM discriminators** |
| --- | --- | --- | --- | --- | --- | --- |
| Akagunduz 2015^40^ | 62 | all | MTV 14ml  SUL_max_ 10.15 | MTV 14ml  SUL_max_ 10.15 | MTV 14ml  SUL_max_ 10.15 | n.s. |
| Castaldi 2012^41^ | 26 | all | n.r. | n.r. | n.r. | n.r. |
| Castelli 2017^42^ | 122 | OPC | n.r. | n.r. | n.r. | n.r. |
| Chen 2014^43^ | 51 | all | n.s. | n.s. | n.s. | n.s. |
| Cheng 2013^44^ | 70 | OPC | n.r. | TLG 121.9 | SUV_max_ 12.87*  TLG 121.9 | n.r. |
| Chung 2009^45^ | 82 | all (77% NPC) | n.r. | n.r. | MTV 40ml | n.r. |
| Higgins 2012^46^ | 88 | all (66% OPC) | n.s. | n.s. | SUV_mean_ 7 | n.s. |
| Katahira-Suzuki 2015^47^ | 70 | all (51% HPC) | SUV_max_ 9.9 | SUV_max_ 9.9* | n.r. | n.s. |
| Kim 2016^48^ | 78 | all (60% OPC) | n.r. | n.r. | n.r. | n.r. |
| Koyasu 2014^49^ | 108 | all | n.r. | n.r. | SUV_max_ 10  MTV 20 ml  TLG 70 | n.r. |
| Lin 2017^50^ | 75 | all (75% OPC) | n.r. | n.r. | n.r. | n.r. |
| Marcus 2014^51^ | 214 | all (63% OPC) | n.r. | n.r. | n.r. | n.r. |
| Matoba 2017^52^ | 40 | all | n.r. | n.r. | n.r. | n.r. |
| Min 2016^53^ | 69 | all (59% OPC) | n.r. | n.r. | n.r. | n.r. |
| Min 2015^54^ | 72 | all (65% OPC) | n.r. | n.r. | n.r. | n.r. |
| Miyabe 2017^55^ | 85 | HPC & LC | n.r. | MTV 28.7 ml | n.r. | MTV 28.7 ml |
| Murphy 2011^56^ | 47 | all | n.r. | n.r. | n.r. | n.r. |
| Ng 2016^53^ | 86 | OPC & HPC | n.r. | SUV_max_ 19.44 | SUV_max_ 19.44  MTV 42.62  TLG 344.72 | n.r. |
| Park 2013^57^ | 81 | HPC & LC (70% LC) | MTV 18 ml | MTV 18 ml | n.r. | n.r. |
| Rasmussen 2015^58^ | 287 | all | n.r. | SUV_max_ 13 | SUV_max_ 13 | n.r. |
| Romesser 2014^59^ | 100 | OPC | MTV 9.7 ml | MTV 9.7 ml | MTV 9.7 ml | MTV 9.7 ml |
| Schwartz 2015^60^ | 74 | all (78% OPC) | SUV_max_ 15.07  MTV 8.76 ml | n.s. | SUV_max_ 15.07  MTV 8.76 ml | n.s. |
| Van den Wyngaert 2017^61^ | 125 | all (55% OPC) | n.r. | n.r. | n.r. | n.r. |
| Yoon 2014^62^ | 40 | NPC | n.r. | MTV 23.01/ 31.45 ml | n.r. | n.r. |
| Zschaeck 2017^63^ | 76 | all | n.r. | n.r. | n.r. | n.r. |

*statistical trend

**Supplementary table 2:** Available clinical data of included patient cohorts

| Sample | # Patients | # SUV | # EFS | # OS | # LRC | # FFDM | # HPV |
| --- | --- | --- | --- | --- | --- | --- | --- |
| All | 1104 | 1002 | 1071 | 1094 | 1088 | 1055 | 277/562 |
| Anderson | 108 | 108 | 108 | 108 | 108 | 108 | 15/76 |
| Charite | 172 | 171 | 171 | 172 | 167 | 166 | 50/66 |
| Qin | 90 | 89 | 81 | 90 | 85 | 83 | 0/58 |
| China | 57 | 57 | 57 | 57 | 57 | 57 | 0/0 |
| Radiomics | 73 | 0 | 73 | 73 | 73 | 73 | 31/35 |
| Brussels | 11 | 11 | 11 | 11 | 11 | 11 | 3/5 |
| Tuebingen | 32 | 22 | 22 | 22 | 28 | 22 | 12/18 |
| Dresden | 87 | 87 | 87 | 87 | 87 | 73 | 12/36 |
| Tcga | 12 | 12 | 0 | 12 | 11 | 0 | 3/5 |
| Poznan | 39 | 39 | 38 | 39 | 38 | 39 | 19/20 |
| Cyprus | 20 | 20 | 20 | 20 | 20 | 20 | 5/9 |
| LMU | 137 | 135 | 137 | 137 | 137 | 137 | 44/56 |
| CHUM | 56 | 55 | 56 | 56 | 56 | 56 | 19/52 |
| CHUS | 95 | 94 | 95 | 95 | 95 | 95 | 32/67 |
| HGJ | 77 | 77 | 77 | 77 | 77 | 77 | 30/43 |
| HMR | 38 | 25 | 38 | 38 | 38 | 38 | 2/16 |

**Table 3:** Patient and tumor characteristics

| Characteristics | Value |
| --- | --- |
| **Age (years)** |  |
| Mean *±* SD Median | 60*±*11  60 |
| **Sex** |  |
| n/a | 8 (0.7%) |
| Male | 874 (79.2%) |
| Female | 222 (20.1%) |
| **T stage** |  |
| n/a | 12 (1%) |
| Tx | 5 (<1%) |
| T1 | 116 (11%) |
| T2 | 281 (26%) |
| T3 | 356 (32%) |
| T4 | 334 (30%) |
| **N stage** |  |
| n/a | 14 (1%) |
| N0 | 225 (20%) |
| N1 | 157 (14%) |
| N2 | 643 (59%) |
| N3 | 65 (6%) |
| **UICC stage** |  |
| n/a | 34 (3%) |
| I | 31 (3%) |
| II | 81 (7%) |
| III | 225 (20%) |
| IV | 733 (67 %) |
| **Localization** |  |
| Other | 19 (2%) |
| Hypopharynx | 110 (10%) |
| Larynx | 186 (17%) |
| Nasopharynx | 141 (13%) |
| Oropharynx | 562 (50%) |
| Oral Cavity | 86 (8%) |
| **Treatment** |  |
| Chemoradiation | 804 (73%) |
| Radiotherapy | 165 (15%) |
| Radiotherapy or chemoradiation | 135 (12%) |

**Supplementary table 4:** Distribution of PET parameters in the whole cohort

| Parameter | Mean *±* SD | Median | IQR | Range |
| --- | --- | --- | --- | --- |
| MTV (ml) | 12.1±16.4 | 7.02 | 3.46 – 15 | 0.001–265 |
| TLG (ml) | 127±194 | 61.6 | 25.2 – 154 | 0.00336–2990 |
| SUV_max_ | 13.9±6.72 | 13 | 9.25 – 17.4 | 0.375–59 |
| SUV_mean_ | 9.02±4.19 | 8.36 | 6.13 – 11 | 0.324–43 |

**Supplementary table 5:** Differences of PET parameters between tumor locations. Shown are p-values resulting from a two-sided t-test.

|  | MTV | TLG | SUV*max* | SUV*mean* |
| --- | --- | --- | --- | --- |
| Hypopharynx vs. Larynx | **0.001** | **0.01** | **0.02** | **0.034** |
| Hypopharynx vs. Nasopharynx | 0.42 | 0.2 | **0.004** | **0.003** |
| Hypopharynx vs. Oropharynx | 0.17 | 0.36 | **0.03** | **0.084** |
| Hypopharynx vs. Oral Cavity | **< 0.001** | **< 0.001** | 0.4 | 0.49 |
| Larynx vs. Nasopharynx | **0.002** | 0.052 | 0.59 | 0.39 |
| Larynx vs. Oropharynx | **< 0.001** | **< 0.001** | 0.49 | 0.36 |
| Larynx vs. Oral Cavity | **< 0.001** | **< 0.001** | **0.004** | **0.0075** |
| Nasopharynx vs. Oropharynx | **0.004** | **< 0.001** | 0.15 | **0.036** |
| Nasopharynx vs. Oral Cavity | **< 0.001** | **< 0.001** | **< 0.001** | **< 0.001** |
| Oropharynx vs. Oral Cavity | **0.002** | **< 0.001** | **0.005** | **0.016** |
| HPV+ vs. HPV- | **0.008** | **0.004** | **0.002** | **0.005** |

**Supplementary table 6:** Univariate Cox regression with respect to EFS, OS, LRC, and FFDM. PET parameters were included as metric parameters. Patients with missing information on one of the parameters/endpoints were excluded from all analyses.

|  |  | **EFS** |  |  | **OS** |  |
| --- | --- | --- | --- | --- | --- | --- |
| Parameter | HR | 95% CI | P-value | HR | 95% CI | P-value |
| Sex male | 1.15 | 0.89 – 1.47 | 0.29 | 1.16 | 0.88 – 1.54 | 0.29 |
| Age *>* 60y | 1.44 | 1.18 – 1.76 | *<* **0.001** | 1.53 | 1.22 – 1.92 | *<* **0.001** |
| T-stage *>* 2 | 1.95 | 1.55 – 2.44 | *<* **0.001** | 2.24 | 1.71 – 2.92 | *<* **0.001** |
| N-stage *>* 0 | 1.23 | 0.95 – 1.61 | 0.12 | 1.53 | 1.11 – 2.12 | **0.0095** |
| UICC-stage *>* III | 1.44 | 1.15 – 1.8 | **0.002** | 1.86 | 1.41 – 2.44 | *<* **0.001** |
| HPC+oral cavity | 2.71 | 2.17 – 3.37 | *<* **0.001** | 3.26 | 2.56 – 4.15 | *<* **0.001** |
| Chemotherapy NO | 1.16 | 0.92 – 1.46 | 0.22 | 1.25 | 0.95 – 1.63 | 0.11 |
| MTV | 1.02 | 1.02 – 1.02 | *<* **0.001** | 1.02 | 1.02 – 1.02 | *<* **0.001** |
| TLG | 1.002 | 1.001 – 1.002 | *<* **0.001** | 1.002 | 1.001 – 1.002 | *<* **0.001** |
| SUVmax | 1.02 | 1.01 – 1.04 | *<* **0.001** | 1.03 | 1.01 – 1.04 | *<* **0.001** |
| SUVmean | 1.03 | 1.01 – 1.05 | **0.003** | 1.04 | 1.01 – 1.06 | **0.002** |
|  |  | **LRC** |  |  | **FFDM** |  |
| Parameter | HR | 95% CI | P-value | HR | 95% CI | P-value |
| Sex male | 0.91 | 0.64 – 1.29 | 0.6 | 1.43 | 0.9 – 2.28 | 0.13 |
| Age *>* 60y | 1.25 | 0.93 – 1.68 | 0.14 | 1.3 | 0.92 – 1.85 | 0.13 |
| T-stage *>* 2 | 2.27 | 1.59 – 3.23 | *<* **0.001** | 1.79 | 1.22 – 2.64 | **0.003** |
| N-stage *>* 0 | 1.12 | 0.76 – 1.65 | 0.57 | 2.67 | 1.44 – 4.95 | **0.002** |
| UICC-stage *>* III | 1.36 | 0.98 – 1.91 | 0.068 | 2.04 | 1.33 – 3.14 | **0.001** |
| HPC+oral cavity | 2.62 | 1.88 – 3.65 | *<* **0.001** | 1.96 | 1.29 – 2.97 | **0.002** |
| Chemotherapy NO | 1.008 | 0.72 – 1.412 | 0.96 | 2.01 | 1.25 – 3.24 | **0.004** |
| MTV | 1.02 | 1.01 – 1.02 | *<* **0.001** | 1.02 | 1.01 – 1.02 | *<* **0.001** |
| TLG | 1.002 | 1.001 – 1.002 | *<* **0.001** | 1.001 | 1.001 – 1.002 | *<* **0.001** |
| SUVmax | 1.02 | 1 – 1.04 | **0.026** | 1.03 | 1 – 1.05 | **0.025** |
| SUVmean | 1.03 | 1 – 1.06 | 0.053 | 1.03 | 1 – 1.07 | 0.074 |

**Supplementary table 7:** Univariate cox regression analyses with respect to EFS, OS, LRC and FFDM. PET parameters were included as binarized parameters.

| Parameter | Risk | HR | 95% CI | p-value |
| --- | --- | --- | --- | --- |
| **EFS** |  |  |  |  |
| MTV | *>* 13.2ml | 2.03 | 1.69 – 2.44 | *<* **0.001** |
| TLG | *>* 154ml | 2.11 | 1.73 – 2.57 | *<* **0.001** |
| SUV_max_ | *>* 15.4 | 1.58 | 1.3 – 1.91 | *<* **0.001** |
| SUV_mean_ | *>* 9.99 | 1.61 | 1.33 – 1.94 | *<* **0.001** |
| **OS** |  |  |  |  |
| MTV | *>* 13.3ml | 2.4 | 1.96 – 2.95 | *<* **0.001** |
| TLG | *>* 154ml | 2.4 | 1.93 – 2.98 | *<* **0.001** |
| SUV_max_ | *>* 14 | 1.58 | 1.28 – 1.95 | *<* **0.001** |
| SUV_mean_ | *>* 9.16 | 1.62 | 1.31 – 1.99 | *<* **0.001** |
| **LRC** |  |  |  |  |
| MTV | *>* 10.4ml | 2 | 1.53 – 2.6 | *<* **0.001** |
| TLG | *>* 180ml | 2.22 | 1.64 – 3.02 | *<* **0.001** |
| SUV_max_ | *>* 8.89 | 2.2 | 1.45 – 3.32 | *<* **0.001** |
| SUV_mean_ | *>* 9.52 | 1.68 | 1.27 – 2.23 | *<* **0.001** |
| **FFDM** |  |  |  |  |
| MTV | *>* 14.9ml | 2.27 | 1.61 – 3.2 | *<* **0.001** |
| TLG | *>* 154ml | 2.13 | 1.49 – 3.05 | *<* **0.001** |
| SUV_max_ | *>* 15.5 | 1.96 | 1.39 – 2.75 | *<* **0.001** |
| SUV_mean_ | *>* 9.32 | 1.92 | 1.37 – 2.69 | *<* **0.001** |

**Supplementary table 8:** cut-off stability testing with minimum and maximum cut-off values leading to significant discrimination of patients.

| Parameter | min. cut-off | opt. cut-off | max. cut-off |
| --- | --- | --- | --- |
| **EFS** |  |  |  |
| MTV | 1.8ml | 13.2ml | 53.3ml |
| TLG | 9.3ml | 154ml | 623.6ml |
| SUV_max_ | 4.8 | 14 | 20.9 |
| SUV_mean_ | 3.3 | 10 | 12.6 |
| **OS** |  |  |  |
| MTV | 1.8ml | 13.3ml | 53.3ml |
| TLG | 9.3ml | 154.1ml | 623.6ml |
| SUV_max_ | 5.7 | 14 | 20.9 |
| SUV_mean_ | 3.8 | 9.2 | 12.2 |
| **LRC** |  |  |  |
| MTV | 1.8ml | 10.4ml | 53.3ml |
| TLG | 10.1ml | 180ml | 623.6ml |
| SUV_max_ | 4.8 | 8.9 | 18.7 |
| SUV_mean_ | 3.4 | 5.7 | 7.8 |
| **FFDM** |  |  |  |
| MTV | 1.9ml | 14.9ml | 31.5ml |
| TLG | 16.3ml | 154.1ml | 340.4ml |
| SUV_max_ | 8.7 | 15.5 | 19.7 |
| SUV_mean_ | 6 | 9.3 | 11.2 |

**Supplementary table 9:** Validation of previously published cut-off parameters by this cohort (taken from supplementary table 1). Publications that only investigated certain tumor locations were only validated by patients with these characteristics (e.g. OPC patients with OPC patients of this study).

| Study | Parameter | LRC | OS | EFS | FFDM |
| --- | --- | --- | --- | --- | --- |
| **All** |  |  |  |  |  |
| Akagunduz 2015 | MTV = 14ml | **<0.001** | **<0.001** | **<0.001** |  |
| Chung 2009 | MTV = 40ml |  |  | **<0.001** |  |
| Higgins 2012 | SUVmean = 7 |  |  | **<0.001** |  |
| Suzuki 2015 | SUVmax = 9.9 | **0.012** | **0.003** |  |  |
| Koyasu 2014 | SUVmax = 10 |  |  | **0.002** |  |
| Koyasu 2014 | MTV = 20ml |  |  | **<0.001** |  |
| Koyasu 2014 | TLG = 70ml |  |  | **<0.001** |  |
| Rasmussen 2015 | SUVmax = 13 |  | **<0.001** | **<0.001** |  |
| Schwartz 2015 | SUVmax = 15.07 | **0.006** |  | **<0.001** |  |
| Schwartz 2015 | MTV = 8.76ml | **<0.001** |  | **<0.001** |  |
| **OPC** |  |  |  |  |  |
| Cheng 2013 | TLG = 121.9ml |  | **<0.001** | **<0.001** |  |
| Cheng 2013 | SUVmax = 12.87 |  |  | **0.011** |  |
| Romesser 2014 | MTV = 9.7 | **<0.001** | **<0.001** | **<0.001** | **0.002** |
| **OPC / HPC** |  |  |  |  |  |
| Ng 2016 | SUVmax = 19.44 |  | 0.43 | 0.27 |  |
| Ng 2016 | MTV = 42.62ml |  |  | **<0.001** |  |
| Ng 2016 | TLG = 344.72ml |  |  | **<0.001** |  |
| **HPC / LC** |  |  |  |  |  |
| Park 2013 | MTV = 18ml | **0.034** | **<0.001** |  |  |
| Miyabe 2017 | MTV = 28.7ml |  | **0.007** |  | **0.029** |
| **NPC** |  |  |  |  |  |
| Yoon 2014 | MTV = 23.01ml |  | **<0.001** |  |  |
| Yoon 2014 | MTV = 31.45ml |  | 0.30 |  |  |

**Supplementary figure 1:** Distribution of PET parameters according to tumor location.


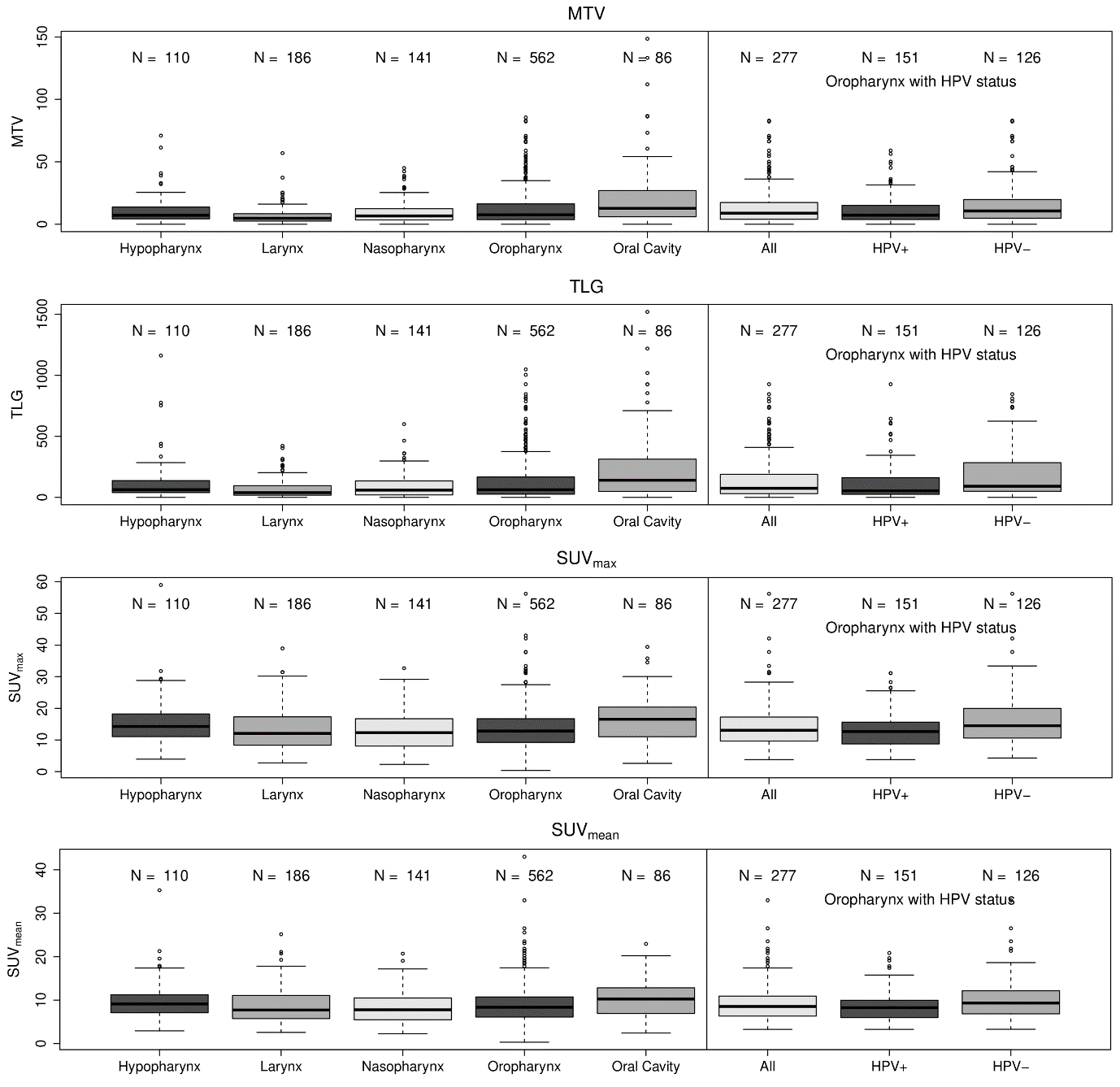


**Supplementary figure 2:** Impact of tumor location on patient outcome in the analyzed cohort.

**
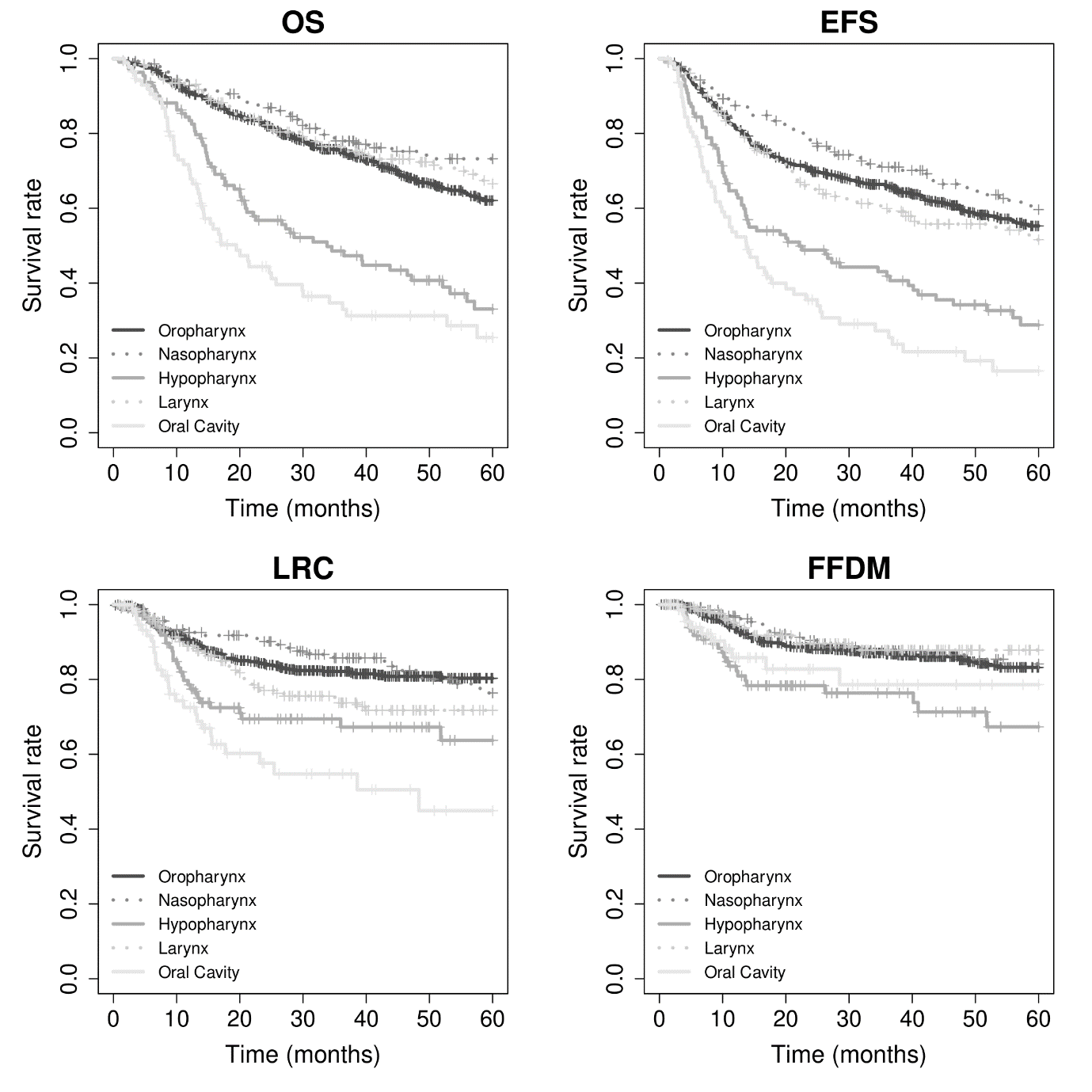
**

**Supplementary figure 3:** Event free survival of all patients when stratified by PET parameters.


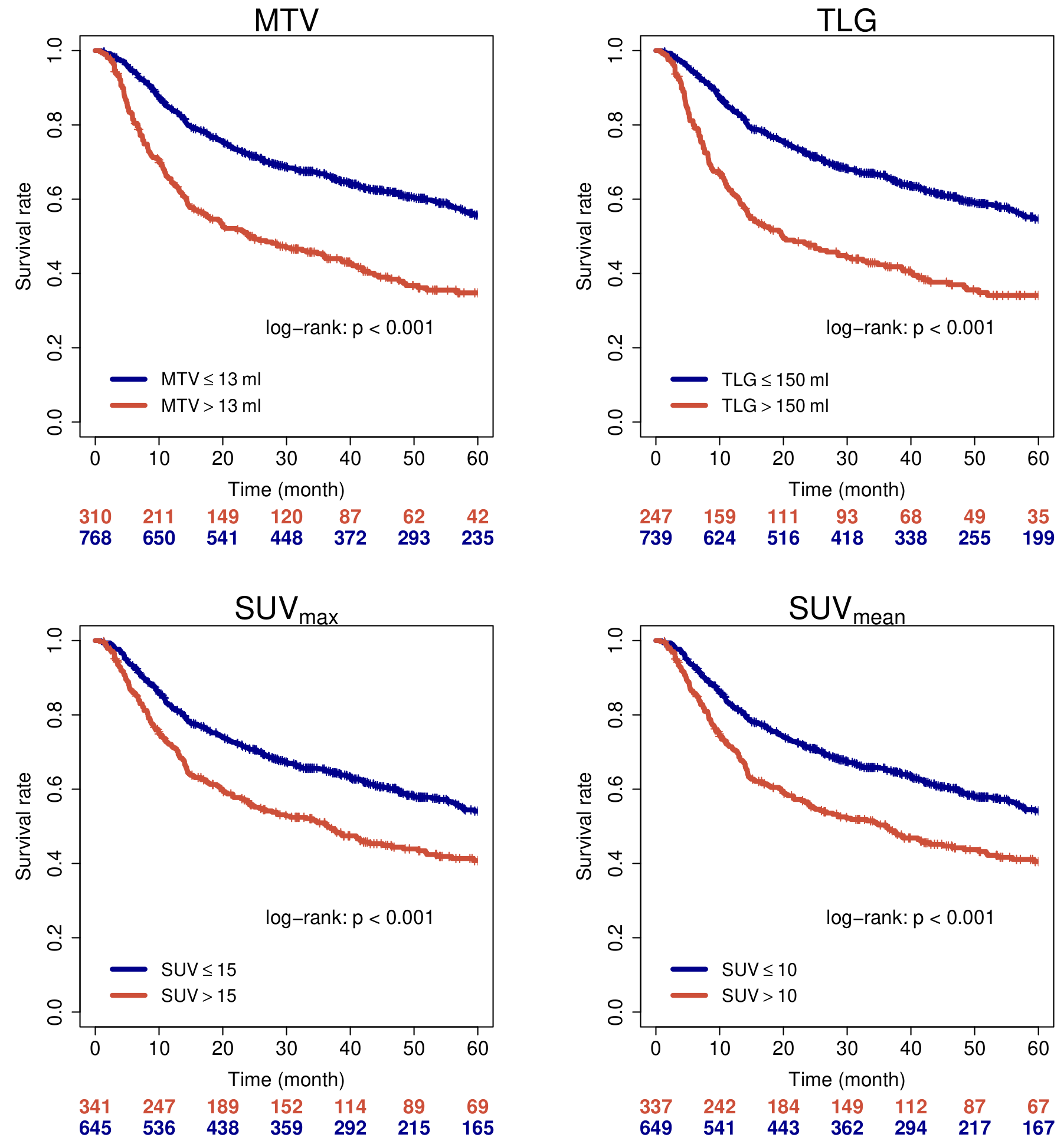


**Supplementary figure 4:** Freedom from distant metastases of all patients when stratified by PET parameters.


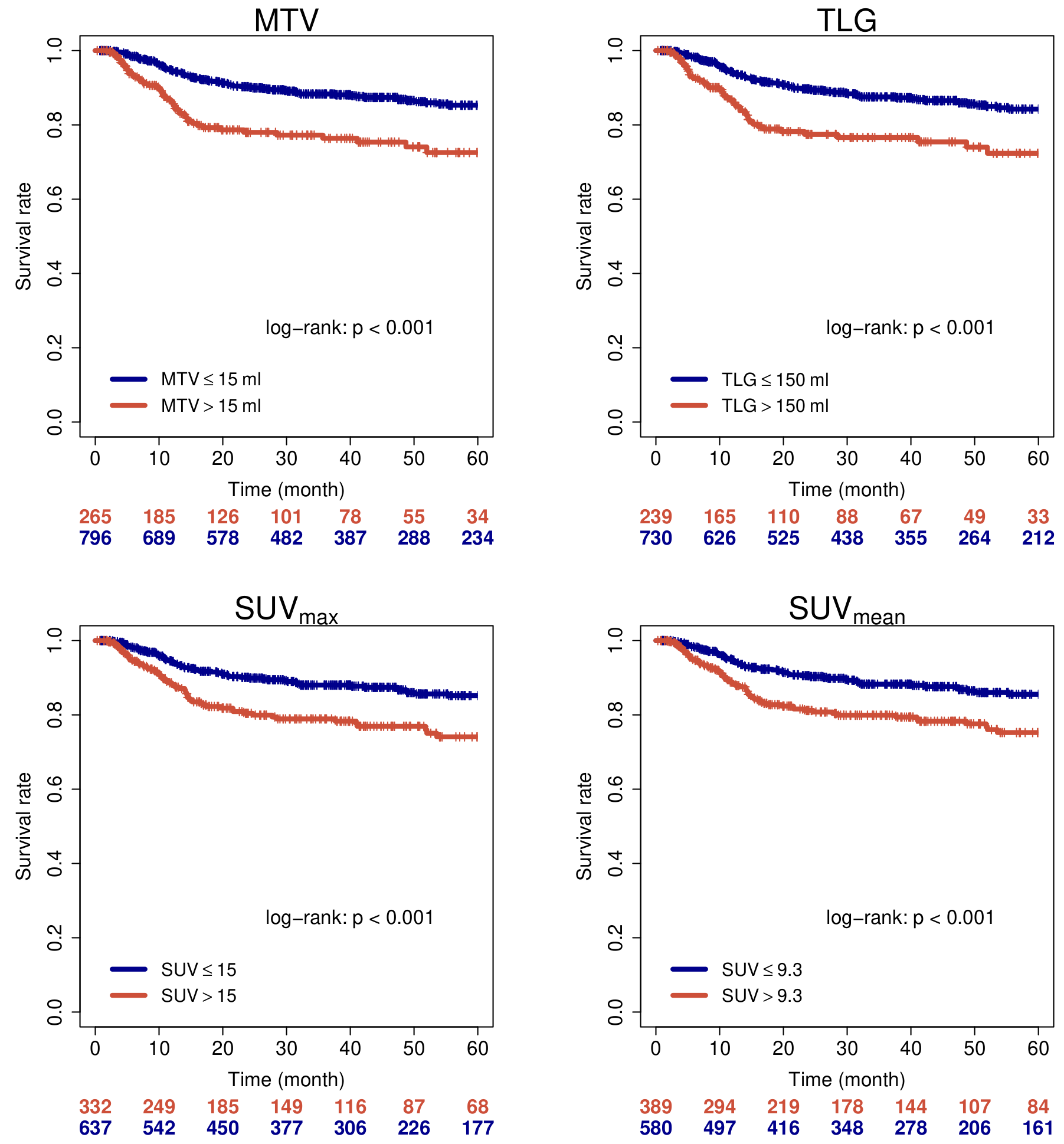


**Supplementary figure 5:** Loco-regional tumor control of laryngeal cancer patients when stratified according to PET parameters.
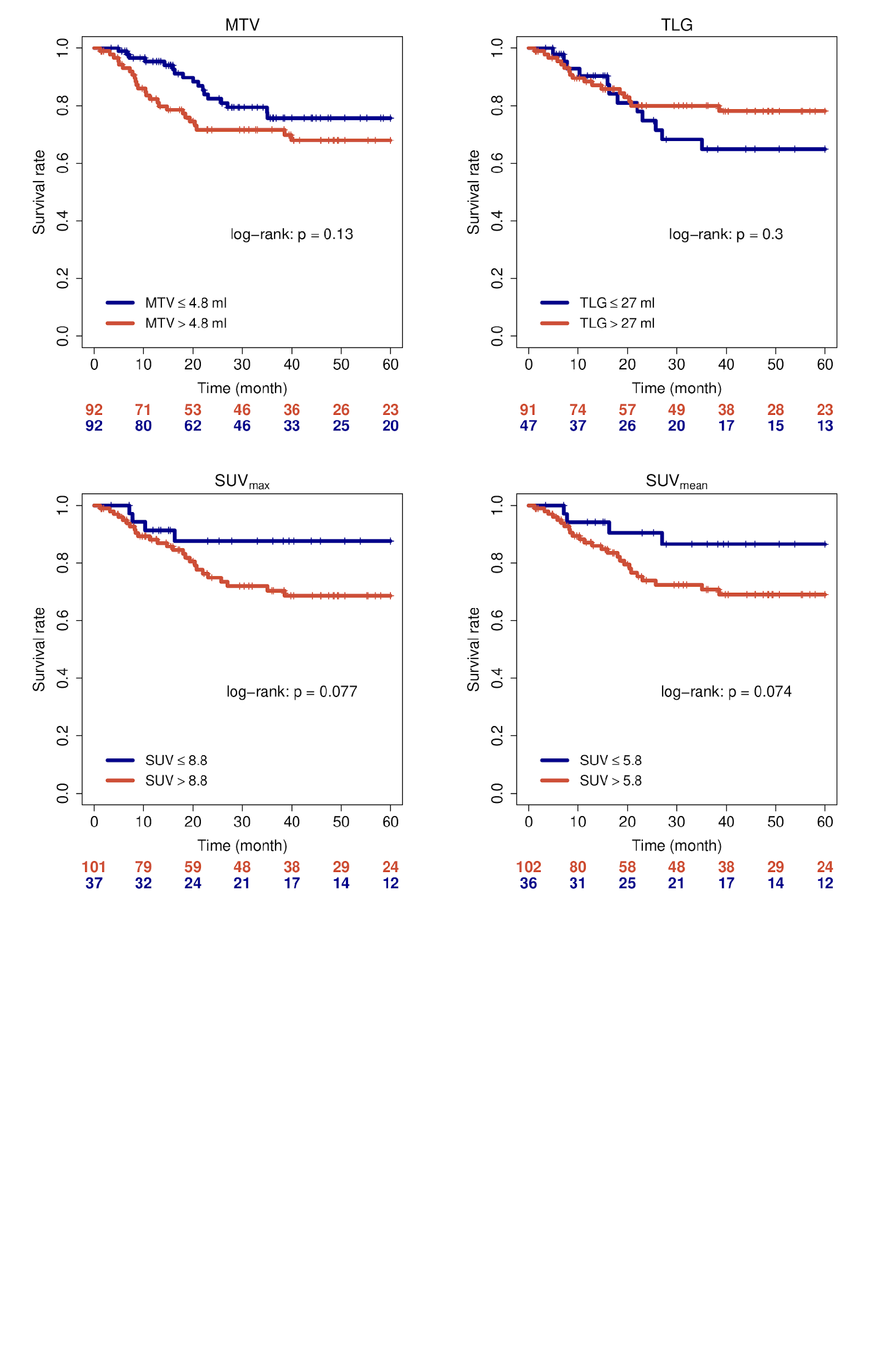

Supplement: Supplementary file 1 [file DataSheet_1.docx]
